# Bisphosphoglycerate mutase predicts myocardial dysfunction and adverse outcome in sepsis: an observational cohort study

**DOI:** 10.1186/s12879-024-09008-6

**Published:** 2024-02-07

**Authors:** Long Huang, Xincai Wang, Bawei Huang, Yu Chen, Xiaodan Wu

**Affiliations:** 1 Department of Critical Care Medicine, Shengli Clinical Medical College of Fujian Medical University, Fujian Provicial Hospital, Fuzhou, China; 2 Medical Department, Shengli Clinical Medical College of Fujian Medical University, Fujian Provicial Hospital, Fuzhou, China; 3 Department of Anesthesiology, Shengli Clinical Medical College of Fujian Medical University, Fujian Provicial Hospital, Fuzhou, China

**Keywords:** Bisphosphoglycerate mutase, Sepsis-induced myocardial dysfunction, Sepsis, Adverse outcome

## Abstract

**Background:**

Sepsis not only causes inflammation, but also damages the heart and increases the risk of death. The glycolytic pathway plays a crucial role in the pathogenesis of sepsis-induced cardiac injury. This study aims to investigate the value of bisphosphoglycerate mutase (BPGM), an intermediate in the glycolytic pathway, in evaluating cardiac injury in septic patients and predicting poor prognosis in sepsis.

**Methods:**

This prospective study included 85 patients with sepsis. Serum BPGM was measured at the time of enrollment, and the patients were divided into a BPGM-positive group (*n* = 35) and a BPGM-negative group (*n* = 50) according to their serum BPGM levels. Baseline clinical and echocardiographic parameters, and clinical outcomes were analyzed and compared between the two groups. Kaplan–Meier analysis was used to compare the 28-day survival rate between BPGM-negative and BPGM-positive patients. Multivariate logistic regression analysis was conducted to explore the independent risk factors for 28-day mortality in septic patients. The predictive value of serum BPGM for sepsis-induced myocardial injury and poor prognosis in sepsis was evaluated using receiver operating characteristic (ROC)curve analysis.

**Result:**

The serum level of BPGM was significantly higher in patients who died within 28 days compared to survivors (*p* < 0.001). Kaplan–Meier analysis showed that serum BPGM-positive sepsis patients had a significantly shorter 28-day survival time (*p* < 0.001). Multivariate logistic regression analysis showed that serum BPGM (OR = 9.853, 95%CI 1.844–52.655, *p* = 0.007) and left ventricular ejection fraction-simpson(LVEF-S) (OR = 0.032, 95% CI 0.002–0.43, *p* = 0.009) were independent risk factors for 28-day mortality in sepsis patients. Furthermore, BPGM levels was negatively correlated with LVEF-S (*p* = 0.005) and positively correlated with the myocardial performance (Tei) index (*p* < 0.001) in sepsis patients. ROC curve analysis showed that serum BPGM was a good predictor of septic myocardial injury and 28-day mortality in sepsis patients.

**Conclusion:**

The level of BPGM in the serum of sepsis patients can serve as a monitoring indicator for myocardial injury, with its high level indicating the occurrence of secondary myocardial injury events and adverse outcomes in sepsis patients.

## Background

Sepsis is life-threatening organ dysfunction caused by a dysregulated host response to infection, affecting millions of people worldwide each year, with a mortality rate of one-sixth to one-third of cases [1, 2]. Organ damage often accompanies sepsis, and in recent years, secondary cardiac damage has gradually gained clinical attention [3]. Clinical studies have shown that 40% of sepsis patients develop sepsis-induced myocardial dysfunction (SIMD), and the mortality rate of these patients can reach 50% to 70% [4–7], which is two to three times higher than that of patients without secondary cardiac damage [8]. Although echocardiography has become a common tool for diagnosing SIMD, the results are still defined ambiguously, and due to its high cost and high operator requirements, continuous dynamic monitoring of echocardiography for each sepsis patient in early resuscitation is impractical [9]. Common myocardial injury indicators such as N-terminal pro-brain natriuretic peptide (NT-proBNP) and high-sensitivity cardiac troponin T (hs-cTnT) lack sufficient specificity in detecting sepsis-induced myocardial disease, making early identification challenging [10]. Therefore, in-depth study of the mechanism of development of SIMD, seeking potential molecular targets for its early diagnosis, prognosis, and treatment, is an urgent issue in clinical practice to alleviate the high mortality rate of SIMD.

Current research suggests that the pathological process of SIMD may involve multiple metabolic pathways, with various factors contributing to the disease, including microcirculatory disorders, myocardial cell metabolic disorders, mitochondrial dysfunction, and calcium ion homeostasis imbalance [10–13]. These factors ultimately lead to impaired myocardial cell metabolism and decreased energy production, in which the glycolytic pathway may play a crucial role in mediating the occurrence and development of SIMD via multiple signaling pathways activated by sepsis. Both domestic and foreign studies have suggested the important regulatory role of the glycolytic pathway in the process of SIMD [14–17]. For instance, Zheng et al. found that immune cells activated by Toll-like receptors (TLRs) can enhance the occurrence of glycolytic metabolism and further regulate the inflammatory response and apoptosis signal transduction to mediate SIMD [18]. Additionally, Pan et al. discovered that the acceleration of glycolysis in septic patients can be suppressed by down-regulating lactate dehydrogenase A (LDHA) of the PI3K/Akt-HIF-1α pathway, which facilitates the immune suppression function of neutrophils and helps to quickly and effectively clear pathogens, thereby improving patient prognosis [19].

The bisphosphoglycerate mutase (BPGM) is a crucial enzyme in the glycolytic pathway, playing an essential role in regulating glucose metabolism. BPGM catalyzes the synthesis and decomposition of 2,3-bisphosphoglycerate (2,3-BPG), which has a unique physiological role in red blood cells by regulating hemoglobin's oxygen affinity and playing an essential role in cellular oxygen transport and release [20]. In sepsis, cellular hypoxia is a common phenomenon due to infection and inflammation leading to tissue edema, vasodilation, and blood stasis, accelerating cell glycolysis, and ultimately causing lactate accumulation and metabolic acidosis, leading to multiple organ failure and death [21, 22]. Although BPGM plays a crucial role in modulating 2,3-BPG concentrations, with indirect effects on the glycolytic pathway, current research primarily focuses on its implications in oncology. Notably, in malignancies such as hepatocellular carcinoma and cervical cancer, BPGM espression is higher in tumor cells than in normal cells [23, 24]. Recent research by Brendon et al. identified BPGM as a potential prognostic marker in sepsis by associating high BPGM expression level with a poor prognosis in patients with the Mars1 molecular phenotype [25]. However, the specific role and mechanism by which BPGM's modulation of 2,3-BPG concentrations influences sepsis, including its potential involvement in SIMD, merit further exploration.

The aim of this study is to investigate the levels of BPGM in the serum of sepsis patients, and to analyze the relationship between the serum BPGM levels and their cardiac injury and adverse outcomes. The study aims to elucidate the role of serum BPGM levels in the evaluation of cardiac injury and prognosis in sepsis patients, and to provide a theoretical basis for serum BPGM as a potential prognostic marker for sepsis.

## Methods

### Study design

We conducted a prospective, observational, single-center cohort study at the Fujian Provincial Hospital South Branch, China, from October 1, 2021 to November 1, 2022. All adult patients (≥ 18 years old) registered with sepsis were included in the analysis. This study was approved by the Ethics Committee of Fujian Provincial Hospital (research code K2021-04–079) and required informed consent from patients and their families.

### Definition of sepsis

According to the third international consensus definition for sepsis and septic shock (Sepsis-3) [26], sepsis is a life-threatening organ dysfunction caused by a dysregulated host response to infection, with clinical diagnosis indicated by a Sequential Organ Failure Assessment (SOFA) score of two or more.

In this study, sepsis was defined according to Sepsis-3.0 criteria and inclusion criteria were age of 18 years or older. Exclusion criteria encompassed patients without ischemic heart disease events such as coronary artery disease or acute myocardial infarction within the past five years, those with no history of chronic heart failure, and patients with indistinct cardiac ultrasonography images.

### Clinical design

#### Data collection

Patient clinical data was recorded, including gender, age, history of underlying diseases such as hypertension, diabetes, coronary heart disease, and chronic obstructive pulmonary disease. All enrolled patients received adequate fluid resuscitation, antimicrobial therapy, and, if necessary, vasopressor support and organ function support treatment according to the sepsis guidelines upon admission. Blood lactate, procalcitonin (PCT), lactate dehydrogenase, anion gap, serum creatinine, creatine kinase, creatine kinase isoenzymes, N-terminal-probrain natriuretic peptid (NT-proBNP), and cardiac troponin I (cTnI) were measured and recorded. Sequential Organ Failure Assessment (SOFA) and Acute Physiology and Chronic Health Evaluation II (APACHE II) scores were evaluated within 24 h. Follow-up was conducted for 28 days through telephone interviews to evaluate mortality rates.

#### BPGM detection

We assessed the serum BPGM levels of all sepsis patients on admission to the study and stored the samples at -80 °C until further analysis. To perform the test, we placed the serum samples at room temperature for 30 min, followed by centrifugation at 3,000 rpm for 15 min at 4 °C. The study utilized an enzyme-linked immunosorbent assay (ELISA) kit provided by Cloud-Clone Corp. (Wuhan, China), with the catalog number SED716Hu, to measure BPGM levels in the collected serum samples. According to the manufacturer's instructions, we considered BPGM levels ≥ 0.312 ng/ml as positive, and those with levels below 0.312 ng/ml as negative.

### Echocardiographic measurements

Upon admission, all sepsis patients were evaluated by experienced cardiologists and/or ultrasound physicians using the EDGE Color Doppler Ultrasound System (Sonosite Inc.). Chest parasternal long-axis and short-axis views, apical 4- and 2-chamber views were obtained according to the guidelines of the American Society of Echocardiography. Baseline echocardiographic parameters, including left ventricular ejection fraction (LVEF) measured by the improved Simpson method, were recorded. LVEF less than 50% was defined as left ventricular systolic dysfunction [9]. The isovolumic contraction time (ICT), isovolumic relaxation time (IRT), and ejection time(ET) were measured. The left ventricular Tei index, a time interval index derived from Doppler echocardiography, was defined as the sum of ICT and IRT divided by ET. The formula for calculating the left ventricular Tei index was as follows: Left ventricular Tei index = ((ICT + IRT)) / ET. The measurements were repeated three times, and the average value was used to correlate the admission BPGM level with cardiac dysfunction observed on echocardiography.

### Statistical analysis

The data analysis was performed using SPSS 26.0 and Graphpad Prism 9.0 statistical software packages. Normality tests were conducted on all quantitative data, Non-normally distributed quantitative data were presented as medians (interquartile range) [M(IQR)] and analyzed using the Mann–Whitney U test. Categorical data were presented as percentages (%) and analyzed using the chi-squared test. Spearman correlation analysis was used to evaluate the linear relationship between non-normally distributed continuous variables. Univariate and multivariate logistic regression analyses were employed to explore the risk factors for 28-day mortality in sepsis, with the calculation of odds ratios (ORs) and 95% confidence intervals (95% CIs). Variables demonstrating a probability of *P* > 0.1 in univariate analyses were excluded from subsequent multivariate logistic regression.The predictive efficiency of BPGM for septic myocardial dysfunction and sepsis-related 28-day mortality was analyzed using receiver operating characteristic (ROC) curves. The relative risk was presented as odds ratio (OR) with 95% confidence intervals (CI). The area under the curve (AUC) was calculated and compared using Graphpad Prism 9.0 software. Kaplan–Meier survival analysis (Log-Rank test) was used to evaluate the survival of the BPGM negative and positive groups. Statistical significance was defined as *P* < 0.05.

## Results

### Characteristics of patients with different serum BPGM levels

**Fig. 1:**
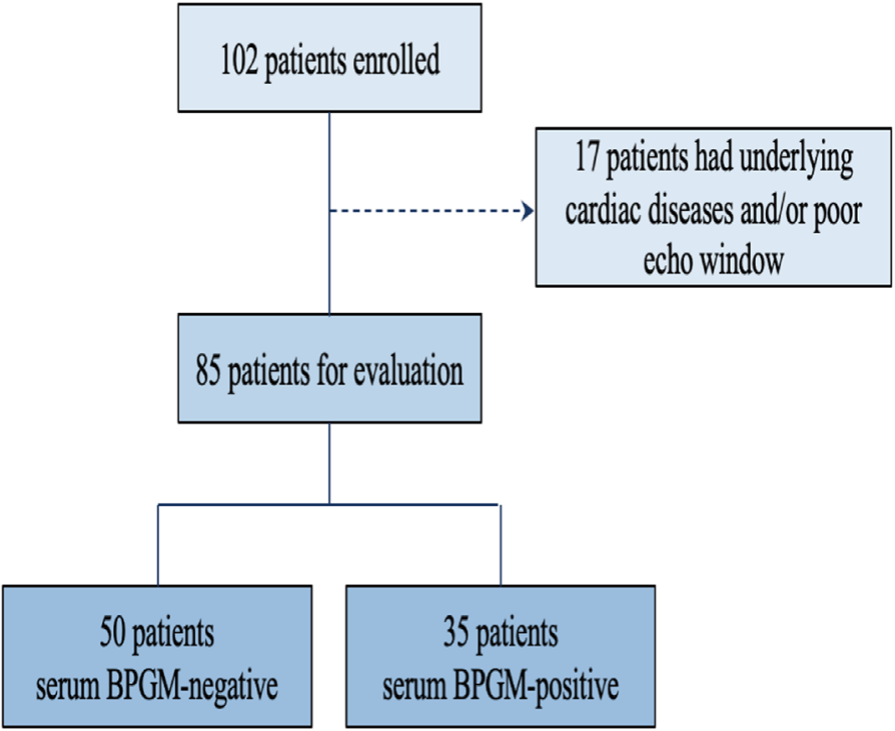
Flow chart of the patients with sepsis in the study

In this clinical study conducted at our center to investigate the predictive value of BPGM in sepsis prognosis, 102 patients who met the inclusion criteria were enrolled. Seventeen patients (16.67%) were excluded due to pre-existing cardiac diseases and/or suboptimal cardiac ultrasound images. Ultimately, 85 patients met the criteria for further evaluation (Fig. 1). Among them, 35 patients (41.18%) with positive serum BPGM were included in the BPGM positive group, while 50 patients (58.82%) with negative serum BPGM levels were included in the BPGM negative group. The baseline characteristics and echocardiographic parameters of the two groups are presented in Table 1.

**Table 1:** Baseline clinical biochemical characteristics (n(%)/M(IQR))

Baseline Parameters	Expression of serum BPGM		*P* value
	**Negative *n* = 50**	**Positive *n* = 35**	
Gender			
Male	33 (66.0)	22 (62.9)	0.765
Female	17 (34.0)	13 (37.1)	
Age, years	68 (20)	64 (24)	0.951
APACHE II, score	23 (9)	27 (12)	**0.022**
SOFA, score	7 (2)	7 (2)	0.924
Comorbidities			
Diabetes Mellitus	16 (32.0)	11 (31.4)	0.956
Immunosuppression	0 (0.0)	1 (2.9)	0.412
IHD	0 (0.0)	2 (1.2)	0.167
Hypertension	25 (50.0)	14 (40.0)	0.363
COPD	2 (4.0)	2 (5.7)	0.713
CKD	4 (8.0%)	4 (11.4)	0.824
Lactate, mmol/l	3.2 (2.0)	3.4 (3.5)	0.165
PCT, ng/ml	27.6(59.7)	28(89.0)	0.422
LDH, IU/l	341 (472)	358 (466)	0.432
AG, mmol/l	18 (8)	17.8 (10)	0.782
CK, IU/l	203 (338.5)	134 (862)	0.486
CK-MB, ng/ml	25 (28)	26 (33)	0.652
NT-proBNP, pg/ml	3440 (4156)	4834 (5419)	0.114
cTnI, ng/ml	0.04 (0.30)	0.18 (0.98)	**0.033**
LVEF-S,%	50 (6.0)	45 (13)	**0.001**
Tei Index	0.39 (0.17)	0.62 (0.24)	**0.001**

Data are presented as a number (%) or median with interquartile ranges. Bold values indicate *P* < 0.05 by Mann–Whitney U test (quantitative data), chi-square (categorical variables) method

*BPGM* bisphosphoglycerate mutase, *APACHE II* Acute Physiology and Chronic Health Evaluation II score, *SOFA* Sequential Organ Failure Assessment, *IHD* ischemic heart disease, *COPD* chronic pulmonary disease, *CKD* chronic kidney disease, *cTnI* cardiac troponin I, *PCT* procalcitonin, *LDH* Lactate dehydrogenase, *AG*, Anion gap, *CK* Creatine kinase, *CK-MB* Creatine kinase-MB, *NT-proBNP* N-terminal-probrain natriuretic peptide, *LVEF-S* left ventricular ejection fraction-simpson, *Tei Index* myocardial performance index, *IQR* interquartile range

The baseline characteristics of the BPGM negative and positive groups were compared, and statistically significant differences were observed in APACHE II (23 vs 27; *p* = 0.022) and cTnI (0.04 vs 0.18; *p* = 0.033) as shown in Table 1. Cardiac color Doppler ultrasound examination of the BPGM negative and positive groups revealed statistically significant differences in LVEF (Simpson's method) (50 vs 45; *p* < 0.01) and Tei index (0.39 vs 0.62; *p* < 0.01) (Table 2), indicating poorer heart function in the BPGM positive group compared to the BPGM negative group.

**Table 2:** Clinical outcomes by different levels of BPGM (n(%)/M(IQR)

	**Expression of serum BPGM**		***P* value**
	**Negative *n* = 50**	**Positive *n* = 35**	
Mechanical ventilation			
Yes	6 (12.0)	7 (20.0)	0.367
No	44 (88.0)	28 (80.0)	
28-day death			
Yes	5 (10.0)	19 (54.3)	** < 0.001**
No	45 (90.0)	35 (35.0)	

Data are presented as a number (%) or median with interquartile ranges. Bold values indicate *P* < 0.05 by chi-square (categorical variables) method

*BPGM* bisphosphoglycerate mutase

After comparing the outcomes between the BPGM negative and positive groups, we found no significant difference in the use of mechanical ventilation after admission (6 vs. 7; *p* = 0.367). However, the 28-day mortality in the positive group was significantly higher than that in the negative group (5 vs. 19; *p* < 0.001), indicating poor prognosis in sepsis patients with high serum BPGM levels.

### Serum BPGM positivity is an independent risk factor for 28-day mortality in septic patients

To objectively evaluate the value of serum BPGM in predicting poor outcomes in septic patients, we divided all patients into a death group (*n* = 24) and a survival group (*n* = 61) based on whether they survived for 28 days. This study conducted a comparative analysis of baseline factors that may affect the mortality outcomes of patients with sepsis (Table 3). Additionally, multivariate logistic regression analysis in this study demonstrated that serum BPGM (adjusted odds ratio [aOR] = 9.853, 95% CI 1.844–52.655, *p* = 0.007) and LVEF-S ([aOR] = 0.032, 95% CI 0.002–0.43, *p* = 0.009) are independent predictors of 28-day mortality in patients with sepsis (Table 4).

**Table 3:** Comparative analysis of 28-day outcome in patients with sepsis (n(%))

Baseline Parameters	28-day outcome		*P* value
	**Survival *n* = 61**	**28-day death *n* = 24**	
Gender			
Male	41 (67.2)	14 (58.3)	0.460
Female	20 (32.8)	10 (41.7)	
Age, years	65 (26)	74 (18)	**0.013**
APACHE II, score	22 (7)	34 (7)	**< 0.001**
SOFA, score	4 (2)	7 (3)	0.636
BPGM, ng/ml			
Negative	45 (73.8)	5 (20.8)	**< 0.001**
Positive	16 (26.2)	19 (79.2)	
Lactate, mmol/l	3.2 (2.1)	3.6 (2.8)	0.136
PCT, ng/ml	20.8(55)	43(81)	0.064
LDH, IU/l	328 (455)	461 (555)	0.133
AG, mmol/l	17.6 (7.6)	19.9 (10.1)	0.488
CK, IU/l	185 (403)	149 (859)	0.938
CK-MB, ng/ml	24 (31)	27 (32)	0.522
NT-proBNP, pg/ml	3456 (4059)	4810 (7442)	0.084
cTnI, ng/ml	0.038(0.19)	0.529 (1.92)	**< 0.001**
LVEF-S,%	52 (6)	36 (8)	**< 0.001**
Tei Index	0.38 (0.15)	0.65 (0.05)	**< 0.001**

Data are presented as a number (%) or median with interquartile ranges. Bold values indicate *P* < 0.05 by Mann–Whitney U test (quantitative data), chi-square (categorical variables) method

*APACHE II* Acute Physiology and Chronic Health Evaluation II score, *SOFA* Sequential Organ Failure Assessment, *BPGM* bisphosphoglycerate mutase, *PCT* procalcitonin, *LDH* Lactate dehydrogenase, *AG* Anion gap, *CK* Creatine kinase, *CK-MB* Creatine kinase-MB, *NT-proBNP* N-terminal-probrain natriuretic peptide, *cTnI* cardiac troponin I, *LVEF-S* left ventricular ejection fraction-simpson, *Tei Index* myocardial performance index, *IQR* interquartile range

**Table 4:** Univariate and multivariate Logistic analysis of 28-day mortality in patients with sepsis

Variables	Univariate analysis		Multivariate analysis	
	***OR*(95%*CI*)**	***P***	***Adjusted***^**a**^*** OR*(95%CI)**	***P***
Gender				
Male	1 (reference)	-		
Female	0.68 (0.26–1.8)	0.442		
Age, years				
< 65	1 (reference)	-		
≥ 65	1.11 (0.43–2.89)	0.828		
APACHE II, score				
< 24	1 (reference)	-	1 (reference)	-
≥ 24	18.17 (3.91–84.55)	**< 0.001**	2.60(0.300–22.546)	0.386
SOFA, score				
< 7	1 (reference)	-		
≥ 7	1.49 (0.55–3.99)	0.433		
BPGM, ng/ml				
< 0.312	1 (reference)	-	1 (reference)	-
≥ 0.312	10.69 (3.42–33.36)	**< 0.001**	9.853(1.844–52.655)	**0.007**
Lactate, mmol/l				
< 3.3	1 (reference)	-		
≥ 3.3	2.21 (0.82 ~ 5.92)	0.116		
PCT, ng/ml				
< 0.5	1 (reference)	-		
≥ 0.5	0.78 (0.07–9.02)	0.842		
LDH, IU/l				
< 246	1 (reference)	-		
≥ 246	2.14 (0.7–6.54)	0.18		
AG,mmol/l				
< 18.5	1 (reference)	-		
≥ 18.5	2.02 (0.77–5.26)	0.152		
CK, IU/l				
< 201	1 (reference)	-		
≥ 201	0.76 (0.29–1.99)	0.571		
CK-MB, ng/ml				
< 31	1 (reference)	-		
≥ 31	1.4 (0.54–3.63)	0.492		
NT-proBNP, pg/ml				
< 900	1 (reference)	-		
≥ 900	5.06 (0.62–41.53)	0.131		
cTnI, ng/ml				
< 0.075	1 (reference)	-	1 (reference)	-
≥ 0.075	6.28 (2.06–19.11)	**0.001**	1.024(0.115–9.073)	0.978
LVEF-S,%				
< 48	1 (reference)	-	1 (reference)	-
≥ 48	0.01 (0–0.07)	**< 0.001**	0.032(0.002–0.43)	**0.009**
Tei Index				
< 0.48	1 (reference)	-	1 (reference)	-
≥ 0.48	43.81 (5.52–347.4)	**< 0.001**	4.319(0.201–92.751)	0.35

Bold values indicate *P* < 0.05

*APACHE II* Acute Physiology and Chronic Health Evaluation II score, *SOFA* Sequential Organ Failure Assessment, *BPGM* bisphosphoglycerate mutase, *PCT* procalcitonin, *LDH* Lactate dehydrogenase, *AG* Anion gap, *CK* Creatine kinase, *CK-MB *Creatine kinase-MB,* NT-proBNP* N-terminal-probrain natriuretic peptide, *cTnI* cardiac troponin I, *LVEF-S* left ventricular ejection fraction-simpson, *Tei Index* myocardial performance index, *IQR* interquartile range

^a^Adjusted for all other variables in model

### The serum BPGM levels is a reliable indicator to assess cardiac dysfunction and prognosis in sepsis patients

To further confirm that patients with high serum BPGM levels are more likely to have myocardial damage (left ventricular dysfunction), we analyzed the correlation between BPGM and cardiac ultrasound parameters LVEF-S and Tei index, and evaluated the efficacy of serum BPGM in predicting cardiac function and 28-day mortality in sepsis patients. As shown in Fig. 3A, sepsis patients with LVEF-S < 50% had higher serum BPGM levels than those with LVEF-S ≥ 50% (*p* < 0.001). Correlation analysis revealed a linear correlation between serum BPGM levels and Tei index (*r* = 0.604, *p* < 0.001, as shown in Fig. 3B), suggesting a negative correlation between BPGM levels and left ventricular systolic function in sepsis patients. ROC curve analysis showed that serum BPGM, as a predictor for LVEF-S < 50%, had a sensitivity of 87.5% and specificity of 77.78% for predicting septic cardiomyopathy when BPGM = 0.62 ng/ml, with an AUC value of 0.838 (*p* < 0.001, 95% confidence interval: 0.707–0.969, standard error: 0.069) (Fig. 3C). These findings indicate that sepsis patients with high serum BPGM levels are more likely to have myocardial damage.

**Fig. 2:**
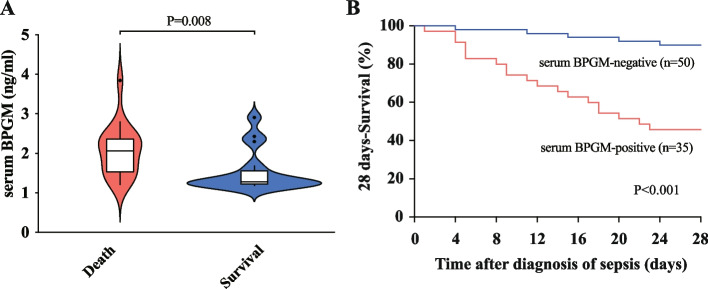
**A** Serum BPGM levels between the 28-day mortality group and the survival group of patients were shown using a violin plot. **B** Kaplan–Meier analysis of the 28-day survival rate of sepsis patients with serum BPGM positive and negative groups

**Fig. 3:**
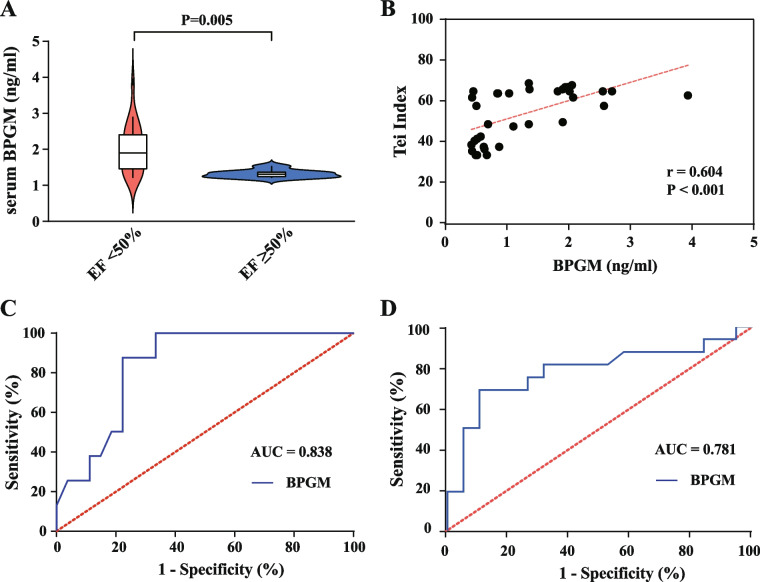
**A** Serum BPGM levels in septic patients with LVEF-S < 50% and LVEF-S ≥ 50% were shown using a violin plot. **B** Spearman’s rank correlation between the serum BPGM levels and Tei index in septic patients. **C** Serum BPGM ROC curve predicting cardiac function in septic patients. **D** Serum BPGM ROC curve predicting 28-day mortality in septic patients

In addition, as shown in Fig. 2A, the serum BPGM levels was significantly higher in patients who died within 28 days compared to those who survived (79.2% vs. 26.2%, *p* < 0.001). The differences were statistically significant, and Kaplan–Meier analysis showed that the serum BPGM-positive sepsis patients had a significantly shorter 28-day survival time (*p* < 0.001, Fig. 2B). The ROC curve analysis for serum BPGM positivity as a predictor of death showed an area under the curve of 0.781 (*p* = 0.005, 95% confidence interval 0.615–0.947, standard error 0.085) (Fig. 3D). These results indicate that high serum BPGM levels suggest a poorer prognosis for sepsis patients and that BPGM levels has good predictive value for sepsis-related 28-day mortality.

## Discussion

Sepsis is a common disease with high mortality [1, 2], and cardiac complications during sepsis are associated with a poor prognosis [27]. Despite years of research, the cellular mechanisms underlying the systolic dysfunction during sepsis remain unclear, and there is currently no effective treatment for sepsis-induced cardiomyopathy [11]. Recent studies have found that the glycolytic pathway plays an important role in the pathological process of secondary cardiac injury during sepsis [14–17]. Exploring the role of the glycolytic intermediate BPGM in cardiac injury in septic patients holds great clinical value.

In this study, we investigated the characteristics of septic patients with different serum BPGM levels in order to identify a new biomarker for early diagnosis and treatment of sepsis. Our findings suggest that serum BPGM positivity is an independent risk factor for sepsis-related 28-day mortality and that the level of serum BPGM levels is a useful indicator for evaluating cardiac function impairment and prognosis in sepsis patients. These results may help predict the severity of sepsis and myocardial injury at an early stage and guide the development of targeted interventions from a more comprehensive perspective.

In previous studies, NT-proBNP, cTnI, or cTnT have been commonly used as auxiliary tools for diagnosis and prognosis of SIMD [9, 28], but whether myocardial dysfunction induces the release of muscle calcium proteins remains a controversial issue. Røsjø and colleagues evaluated high-sensitivity cardiac troponin T (hs-cTnT) in a large population of sepsis patients and found that hs-cTnT levels only reflected myocardial cell injury and could not reliably identify SIMD [29]. Masson and others reported that both NT-proBNP and hs-cTnT lack sufficient specificity for detecting sepsis-induced myocardial disease and are inadequate as diagnostic markers [30]. In contrast, our study demonstrated that a positive serum BPGM predicted sepsis-induced myocardial disease with an area under the ROC curve of 0.838, and when BPGM was 0.62 ng/ml, it had a sensitivity of 87.5% and specificity of 77.78% in predicting sepsis-induced myocardial disease. This suggests that an elevated BPGM level can well predict sepsis-induced myocardial injury. Additionally, we found that the 28-day survival time of sepsis patients with positive serum BPGM was significantly shorter, indicating good predictive efficacy for poor prognosis of sepsis (AUC = 0.781). This further confirms that sepsis-induced myocardial injury is often associated with poor prognosis. According to previous studies, BPGM is an important intermediate in regulating the glycolytic process, which can directly regulate the generation of 1,3-BPG to 2,3-BPG, affecting the glycolysis rate, and may affect the energy metabolism of sepsis [31]. However, this mechanism has not been studied yet. Our study suggests that the over-activation of BPGM in sepsis patients may mediate the disturbance of the glycolytic pathway, leading to a series of adverse reactions such as myocardial cell mitochondrial aerobic oxidation deficiency, exacerbating tissue acidosis and hypoxia, and worsening the patient's condition. Sun and others showed that BPGM levels was significantly upregulated in a state of hypoxia along with an increase in cellular glycolysis levels [20], indicating that BPGM may play an important role in the process of sepsis glycolysis.

In this study, we found that the levels of BPGM in patients' serum is significantly correlated with cardiac functional indices such as LVEF-S and the left heart Tei index, and may serve as a good substitute for echocardiographic parameters, cTnI, and NT-proBNP for diagnosing septic myocardial injury. Currently, the clinical definition and diagnosis of SIMD have not been fully unified [32, 33], as its manifestations of cardiac dysfunction on echocardiography are diverse and complex [34, 35]. Typical parameters such as LVEF, left ventricular end-diastolic volume (LVEDV), and left heart Tei, which are significantly associated with hypotension and cardiac injury, are commonly used as diagnostic indicators for SIMD [36–38]. Kim et al. used LVEF to evaluate left ventricular systolic dysfunction in septic shock patients and found that although the degree of LVEF decline was nonlinearly correlated with adverse outcomes in septic patients, patients with a decrease in LVEF had significantly higher in-hospital mortality rates [39], which is consistent with our study results. In a prospective cohort study of sepsis patients, improvement in the left heart Tei index 24 h after hospitalization was associated with a lower 90-day mortality rate (17% vs. 47%) [32]. However, echocardiography equipment is often expensive and requires experienced physicians to perform the examination. In resource-limited areas and primary hospitals, there is a lack of both equipment and physicians to perform echocardiography. According to a study in Lancet [40], the incidence and mortality rates of sepsis vary greatly in different regions, with around 85.0% of sepsis cases occurring in middle- and low-income countries [1]. Sub-Saharan Africa, South Asia, and Southeast Asia bear the heaviest burden of sepsis, where disease burden is often high and medical resources are scarce. In comparison, the measurement of serum BPGM levels will be more cost-effective than echocardiography because the measurement only requires a complete ELISA test, which will play a significant role in resource-limited areas.

BPGM is predominantly present in erythrocytes and placental cells, and previous studies have focused primarily on its role in regulating hemoglobin's affinity for oxygen [20, 41]. However, evidence suggests that BPGM may have gene level in various tissues and cells, and could play a central role in the pathogenesis of myocardial injury [42]. In our findings, patients with septic myocardial disease exhibited elevated serum levels of BPGM, indicating an active 2,3-DPG shunt pathway. This activity leads to a reduction in the conversion of 1,3-bisphosphoglycerate to 3-phosphoglycerate and an increase in the conversion of 2,3-DPG to 3-phosphoglycerate, ultimately resulting in decreased ATP production via glycolysis. Consequently, this might contribute to the higher 28-day mortality rate in septic myocardial disease [7], which could denote a distinct aspect of glycolytic pathway alteration compared to sepsis alone, suggesting BPGM as a potential diagnostic or therapeutic target for septic myocardial disease. While further basic and clinical research is needed to confirm this, our study provides a viable pathway for investigating the mechanisms underlying septic myocardial disease.

In addition, our study found that BPGM-positive patients had higher APACHE II scores, indicating that serum BPGM may reflect the severity of clinical and physiological abnormalities in sepsis patients to some extent. We also found that high BPGM levels was often accompanied by myocardial injury, leading to increased heart rate and respiratory frequency beyond the normal range, and hence an inevitable increase in APACHE II scores. This suggests that septic patients with high BPGM levels and concurrent heart failure have a more severe condition, often resulting in prolonged intensive care unite(ICU) stays or poor treatment outcomes [36, 43, 44]. Therefore, when studying sepsis, attention should be paid to common cardiac complications. Early detection, diagnosis, and proactive intervention can effectively improve the prognosis of sepsis patients.

Our study presents several notable limitations. Firstly, it was a single-center study at a tertiary hospital in China, which may introduce a selection bias. Future research should consider multi-center, prospective studies to corroborate our findings. Secondly, the cardiac ultrasound indices utilized were relatively basic, as the left ventricular ejection fraction (LVEF) and the left heart Tei index only reflect systolic function. Subsequent studies could include a broader array of echocardiographic measures to more thoroughly investigate the complex presentations of SIMD. Thirdly, BPGM levels were measured only within the initial 24 h of ICU admission, without subsequent dynamic monitoring, potentially limiting our understanding of its temporal changes during the evolution of sepsis. In addition, the ELISA kit used for BPGM quantification had a detection limit of 0.312 ng/ml, presenting challenges in reliably quantifying levels below this threshold and possibly introducing uncertainty in the measurements of low BPGM levels. This could impact the interpretation of BPGM as a biomarker in sepsis, and we recommend employing more sensitive detection techniques in future studies to address this limitation. Lastly, due to the limited sample size, we were unable to perform subgroup and stratified analyses to further affirm the robustness of our results.

## Conclusion

In conclusion, this study suggests that the serum level of BPGM can be used to monitor myocardial damage in septic patients and is positively correlated with the degree of cardiac dysfunction. High level of serum BPGM indicates the occurrence of myocardial damage events and poor outcomes in septic patients.

## Data Availability

All data generated or used during the study are available from the corresponding author by request.

## Abbreviations

AG: Anion gap
APACHE II: Acute Physiology and Chronic Health Evaluation II score
AUC: Area under the curve
BPG: Bisphosphoglycerate
BPGM: Bisphosphoglycerate mutase
CI: Confidence intervals
CK: Creatine kinase
CKD: Chronic kidney disease
CK-MB: Creatine kinase-MB
COPD: Chronic pulmonary disease
cTnI: Cardiac troponin I
ELISA: Enzyme-linked immunosorbent assay
ET: Ejection time
ICT: Isovolumic contraction time
ICU: Intensive care unit
IHD: Ischemic heart disease
IQR: Interquartile range
IRT: Isovolumic relaxation time
LDH: Lactate dehydrogenase
LDHA: Lactate dehydrogenase A
LVEDV: Ventricular end-diastolic volume
LVEF-S: Left ventricular ejection fraction-simpson
NT-proBNP: N-terminal-probrain natriuretic peptide
OR: Odds ratio
PCT: Procalcitonin
SOFA: Sequential Organ Failure Assessment
TLR: Toll-like receptor
Tei Index: Myocardial performance index
